# Efficacy and safety of telitacicept, a BLyS/APRIL dual inhibitor, in the treatment of IgA nephropathy: a retrospective case–control study

**DOI:** 10.1093/ckj/sfae285

**Published:** 2024-09-13

**Authors:** Meng Wang, Jianfei Ma, Li Yao, Yi Fan

**Affiliations:** Department of Nephrology, First Hospital of China Medical University, Shenyang, Liaoning Province, China; Department of Nephrology, First Hospital of China Medical University, Shenyang, Liaoning Province, China; Department of Nephrology, First Hospital of China Medical University, Shenyang, Liaoning Province, China; Department of Nephrology, First Hospital of China Medical University, Shenyang, Liaoning Province, China

**Keywords:** corticosteroid therapy, estimated glomerular filtration rate, IgA nephropathy, proteinuria, telitacicept

## Abstract

**Background:**

Telitacicept, a B lymphocyte stimulator/A proliferation-inducing ligand dual-target fusion protein, has recently been used in autoimmune diseases. We assessed the efficacy and safety of telitacicept in immunoglobulin A nephropathy (IgAN) patients.

**Methods:**

This study included 42 IgAN patients who received telitacicept treatment, forming the ‘whole telitacicept group’. Among them, 20 patients who had not previously received corticosteroid (CS) therapy or immunosuppressive (IS) agents were categorized as the ‘newly treated telitacicept subgroup’. Additionally, 28 patients who were selected to match historical controls received conventional IS therapy (CS therapy with/without IS agents) and were classified as the ‘conventional IS group’. Telitacicept was partially used in combination with conventional IS therapy, including initial CS in different doses. Various indicators were compared at 4-week intervals up to 24 weeks among the three groups.

**Results:**

After 24 weeks of treatment, the 24-hour proteinuria decreased from 1.70 g [interquartile range (IQR) 1.05–2.58] to 0.21 g (IQR 0.39–0.13) (*P* = .043) in the newly treated telitacicept subgroup, from 1.78 g (IQR 0.97–2.82) to 0.44 g (IQR 1.48–0.16) (*P* = .001) in the conventional IS group and from 1.07 g (IQR 0.66–1.99) to 0.26 g (IQR 0.59–0.17) (*P* = .028) in the whole telitacicept group. The estimated glomerular filtration rate (eGFR) increased from 76.58 ± 30.26 ml/min/1.73 m^2^ to 80.30 ± 26.76 ml/min/1.73 m^2^ (*P* = .016) in the newly treated telitacicept subgroup, from 72.73 ± 33.41 ml/min/1.73 m^2^ to 84.08 ± 26.81 ml/min/1.73 m^2^ (*P* = .011) in the conventional IS group and from 70.10 ± 32.88 ml/min/1.73 m^2^ to 71.21 ± 31.49 ml/min/1.73 m^2^ (*P* = .065) in the whole telitacicept group. During follow-up periods, the efficacy rates of the three groups did not show statistically significant differences and no serious adverse events were observed.

**Conclusions:**

Telitacicept may be a safe and effective treatment for IgAN, offering reductions in proteinuria and increases in eGFR similar to conventional IS therapy. After a 24-week follow-up, the incidence of adverse events was lower for telitacicept than for conventional IS therapy.

KEY LEARNING POINTS **What was known:**

- Telitacicept has passed phase 2 clinical trials for the treatment of immunoglobulin A nephropathy (IgAN). However, there are no reports or data available from real-world studies at present.

**This study adds:**

- Telitacicept achieved similar clinical efficacy in IgAN compared with IS therapy, significantly reducing proteinuria and increasing estimated glomerular filtration rate while exhibiting good safety.
- Telitacicept may be a supplement or alternative treatment for IgAN, helping to avoid or minimize the side effects associated with corticosteroids.

**Potential impact:**

- Telitacicept provides a novel immunotherapy option for IgAN treatment.

## INTRODUCTION

Immunoglobulin A nephropathy (IgAN) is the most common primary glomerular disease worldwide and is a leading cause of chronic kidney disease (CKD) and renal failure in young adults, with significant regional variations [1]. IgAN lacks specific therapeutic drugs. Corticosteroid (CS) therapy and immunosuppressive (IS) agents cannot completely control disease progress. Thus it is essential to seek better therapies for disease control.

Galactose-deficient IgA1 (Gd-IgA1) is one of the main pathogenic factors in IgAN, and the level of Gd-IgA1 is associated with disease progression [2]. Both Gd-IgA1 and anti-Gd-IgA1 autoantibody production are believed to be driven primarily by mucosal plasma cells [3]. B lymphocyte stimulator (BLyS) and A proliferation-inducing ligand (APRIL) binding to receptors on the surface of B cells activate them, initiating the proliferation and differentiation of B cells. This process leads to the production of Gd-IgA1, which contributes to the development of IgAN [2, 4]. Inhibiting BLyS and APRIL can reduce Ig levels and proteinuria in IgAN patients, thus presenting a novel avenue in the exploration of treatment options for IgAN.

Telitacicept, a dual-target receptor-antibody fusion protein, targets both BLyS and APRIL, inhibiting B cell maturation and differentiation in multiple stages. It is administered through weekly subcutaneous (SC) injections and has few adverse reactions. It has been successfully used in the treatment of systemic lupus erythematosus (SLE), showing good therapeutic effects [5–9] and has successfully completed phase 2 clinical trials for the treatment of both primary Sjögren’s syndrome [10] and IgAN [11]. However, there are currently no reports or data available from real-world studies for IgAN.

Based on the above information, telitacicept may improve the prognosis of IgAN patients. Therefore, we designed and conducted a retrospective case–control study to evaluate the efficacy and safety of telitacicept in addition to or instead of conventional IS therapy in the treatment of IgAN.

## MATERIALS AND METHODS

### Patient selection

This study was a single-centre retrospective case–control study. We collected patients who visited the Department of Nephrology at the First Affiliated Hospital of China Medical University from January 2022 to October 2022 who met the inclusion criteria: age 18–65 years, any gender, confirmed primary IgAN by kidney biopsy and treated with telitacicept. Exclusion criteria were secondary IgAN (including but not limited to allergic purpura, SLE, ankylosing spondylitis, liver cirrhosis, infection, etc.), a lack of baseline data or relevant follow-up data, treatment duration <8 weeks and pregnant or lactating women.

### Study design

#### Grouping principle

All patients treated with telitacicept were classified as the ‘whole telitacicept group’ (telitacicept therapy with or without IS therapy). Within the whole telitacicept group, patients who had not previously received CS therapy or IS agents were further categorized as the ‘newly treated telitacicept subgroup’. The control group, classified as the ‘conventional IS group’ (CS therapy with or without IS agents), consisted of patients from the same hospital and time period who received their first IS therapy without telitacicept and matched to the newly treated telitacicept subgroup by age, gender, proteinuria and estimated glomerular filtration rate (eGFR). The differences in various indicators before and after treatment were compared among the three groups. The efficacy differences between the newly treated telitacicept subgroup and the conventional IS group were also evaluated.

#### Data collection

This study collected clinical data from patients, including general information and laboratory test results, at the baseline (based on the last laboratory test before telitacicept or CS treatment) and at 4, 8, 12, 16, 20 and 24 weeks after treatment initiation. Adverse reactions were also recorded. The general information collected included gender, age, body mass index (BMI), vital signs, medical history, complications, therapy regimens and adverse reactions. The laboratory tests included 24-hour proteinuria, urinary red blood cell (RBC) count, haemoglobin (Hgb), serum albumin (ALB) and eGFR (using the Chronic Kidney Disease Epidemiology Collaboration equation [12]).

#### Observational indicator

The primary observational indicators were the changes in 24-hour proteinuria and eGFR after 24 weeks of treatment compared with baseline. The secondary observational indicators were the changes in ALB, Hgb, and urinary RBC count after 24 weeks of treatment compared with baseline. The rates of complete remission (CR), partial remission (PR) and no remission in IgAN patients after 24 weeks of treatment compared with baseline were also evaluated.

#### Efficacy assessment

CR was defined as patients with 24-hour proteinuria ≤0.3 g, ALB >35 g/l and stable renal function (a decrease in eGFR ≤30%). Patients who experienced a >50% reduction in 24-hour proteinuria while maintaining stable renal function but did not achieve CR were classified as PR. Patients who failed to achieve CR or PR after 24 weeks of treatment were categorized as no remission. The overall efficacy rate was calculated by summing the cases of CR and PR, divided by the total number of cases, and multiplied by 100%.

#### Safety assessment

The occurrence of adverse events (AEs) and serious adverse events (SAEs) were carefully assessed. SAEs were defined as adverse reactions with the potential to be life-threatening, result in death, require hospitalization or an extended hospital stay or cause persistent serious organ or functional disorders. The evaluation included injection site reactions, infections at any site, liver damage, reproductive system damage, osteonecrosis or bone fractures, haematological system damage, gastrointestinal system damage, cardio-cerebrovascular disease and other AEs.

### Statistical methods

For continuous variables, normally distributed data were presented as mean ± standard deviation (SD) and non-normally distributed data were presented as median [interquartile range (IQR) 25–75%]. Parametric tests such as *t*-tests and non-parametric tests such as Wilcoxon rank sum tests were used to compare the differences between groups. The chi-squared test and Kaplan–Meier survival analysis were used to evaluate the differences in remission rate among groups. *P*-values <.05 were considered statistically significant.

## RESULTS

### Baseline characteristics

This study included a total of 70 eligible IgAN patients, with 42 individuals in the whole telitacicept group, 20 in the newly treated telitacicept subgroup and the remaining 28 in the conventional IS group. The patients in the whole telitacicept group who had received CS therapy or IS agents before the study had an average disease duration of 3.00 years (IQR 0.50–6.00) before the study. The patients in the newly treated telitacicept subgroup and the conventional IS group were newly diagnosed through biopsy and had not received CS therapy or IS agents before the study. The follow-up periods for each group were 20.00 weeks (IQR 16.00–24.00), 20.00 weeks (IQR 16.00–24.00), and 24 weeks (IQR 20.00–24.00), respectively. All patients received treatment with telitacicept or CS therapy for at least 12 weeks. The baseline characteristics of all patients are shown in Table 1.

**Table 1:** Main clinical and laboratory characteristics at baseline.

Characteristics	Whole telitacicept group (*n* = 42)	Newly treated telitacicept subgroup (*n* = 20)	Conventional IS group (*n* = 28)	*P*-value
Age (years), mean (SD)	37.52 (11.39)	38.15 (11.70)	41.54 (12.80)	.391
Female, *n*(%)	19 (45.2)	8 (40.0)	16 (57.1)	.247
Weight (kg), mean (SD)	77.61 (18.01)	79.65 (19.33)	72.34 (14.20)	.191
BMI (kg/m^2^), mean (SD)	26.80 (4.37)	27.26 (4.50)	26.19 (4.01)	.433
Hypertension, *n* (%)	24 (57.1)	12 (60.0)	17 (60.7)	.961
Systolic blood pressure (mmHg), mean (SD)	133.52 (14.33)	134.20 (13.54)	133.89 (15.32)	.967
Diastolic blood pressure (mmHg), mean (SD)	82.02 (11.77)	82.35 (13.17)	81.75 (11.40)	.900
Diabetes, *n* (%)	6 (14.3)	2 (10)	2 (7.1)	.727
Oxford classification, *n*				
M 0/1	14/28	7/13	7/21	.457
E 0/1	28/14	13/7	18/10	.960
S 0/1	2/40	0/20	1/27	.398
T 0/1/2	14/17/11	8/7/5	9/12/7	.713
C 0/1/2	32/9/1	17/3/0	19/7/2	.157
Previous CS, *n* (%)	16 (38.1)	0 (0)	0 (0)	
Previous IS agents, *n* (%)	15 (35.7)	0 (0)	0 (0)	
Current CS or IS agents, *n* (%)	22 (52.4)	10 (50)	28 (100.0)	<.01
Current CS, *n* (%)	16 (38.1)	8 (40.0)	28 (100)	<.01
Current IS agents, *n* (%)	16 (38.1)	7 (35)	13 (46.4)	.311
Hydroxychloroquine, *n* (%)	31 (73.8)	17 (85)	23 (82.1)	.558
ARB, *n* (%)	23 (54.8)	16 (80.0)	22 (78.5)	.599
Treatment duration (weeks), median (IQR)	16.00 (12.00–24.00)	16.00 (13.00–24.00)	24.00 (20.00–24.00)	
Follow-up period (weeks), median (IQR)	20.00 (16.00–24.00)	20.00 (16.00–24.00)	24.00 (20.00–24.00)	
24-hour proteinuria (g/day), median (IQR)	1.07 (0.66–1.99)	1.70 (1.05–2.58)	1.78 (0.97–2.82)	.770
eGFR (ml/min/1.73 m^2^), mean (SD)	70.10 (32.88)	76.58 (30.26)	72.73 (33.41)	.684
ALB (g/l), median (IQR)	37.65 (35.60–40.75)	37.55 (35.00–40.98)	36.25 (32.38–38.70)	.225
Hgb (g/l), mean (SD)	132.67 (19.79)	133.80 (18.61)	129.36 (20.27)	.433
Urinary RBC count (cells/μl), median (IQR)	35.40 (6.23–137.08)	88.15 (8.85–195.03)	58.45 (17.60–178.28)	.818

*P-*value was the statistical difference between the newly treated telitacicept subgroup and the conventional IS group.

In the whole telitacicept group, only one patient was given 240 mg/week SC injections, while the other 41 patients were given 160 mg/week SC injections. The specific dosages and durations of CS and IS agents used in the three groups are shown in Supplementary data Table S1. The usage rates of angiotensin receptor blocker (ARB) before the study baseline in the whole telitacicept group, the newly treated telitacicept subgroup and the conventional IS group were 47.6%, 65% and 64.3%, respectively. The duration of treatment was 6.00 months (IQR 3.00–12.00), 4.00 months (IQR 3.00–6.00) and 6.00 months (IQR 3.00–10.50), respectively.

### Primary outcomes

**Figure 1:**
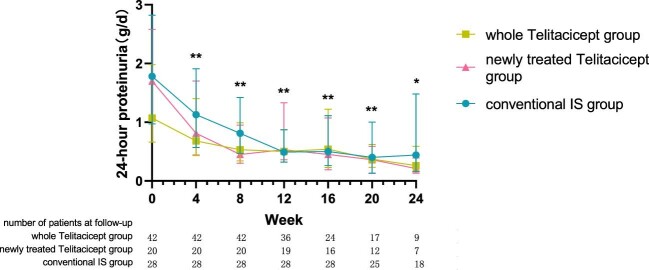
The changes in 24-hour proteinuria level in the whole telitacicept group, the newly treated telitacicept subgroup and the conventional IS group during each follow-up period. The dots represent the median proteinuria values and the bars represent the 25th and 75th percentiles. In the whole telitacicept group, the level of proteinuria was compared with the baseline level every 4 weeks. *P < .05, **P < .01.

**Figure 2:**
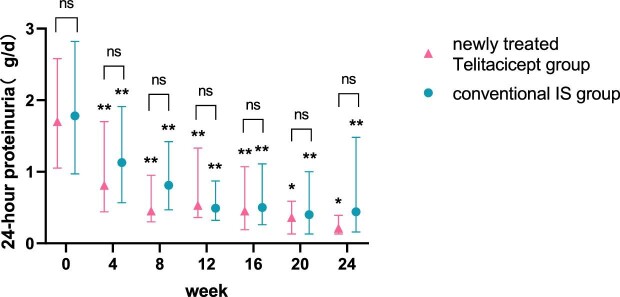
Comparison of 24-hour proteinuria level between the newly treated telitacicept subgroup and the conventional IS group. The dots represent the median proteinuria values and the bars represent the 25th and 75th percentiles. The level of proteinuria was compared with the baseline level every 4 weeks. *P < .05, **P < .01; ns: not significant.

The level of 24-hour proteinuria at 24 weeks decreased from 1.07 g (IQR 0.66–1.99) to 0.26 g (IQR 0.17–0.59) (*P* = .028) in the whole telitacicept group, from 1.70 g (IQR 1.05–2.58) to 0.21 g (IQR 0.13–0.39) (*P* = .043) in the newly treated telitacicept subgroup and from 1.78 g (IQR 0.97–2.82) to 0.44 g (IQR 0.16–1.48) (*P* = .001) in the conventional IS group. There were no statistically significant differences between the newly treated telitacicept subgroup and the conventional IS group in terms of 24-hour proteinuria at baseline (*P* = .770), 4 weeks (*P* = .408), 8 weeks (*P* = .156), 12 weeks (*P* = .330), 16 weeks (*P* = .657), 20 weeks (*P* = .516) and 24 weeks (*P* = .126) (Figs. 1 and 2).

**Figure 3:**
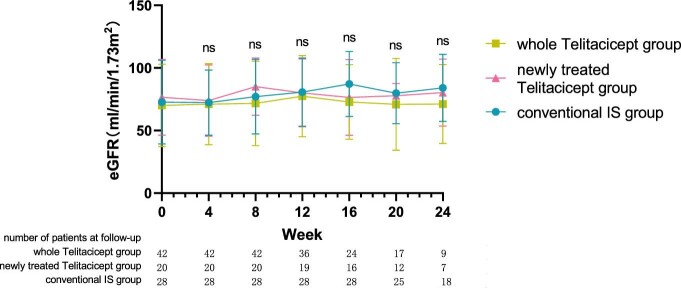
The changes in eGFR level in the whole telitacicept group, the newly treated telitacicept subgroup and the conventional IS group during each follow-up period. The dots represent the mean value of eGFR and the bars represent the SD. In the whole telitacicept group, the level of eGFR was compared with the baseline level every 4 weeks. *P < .05, **P < .01; ns: not significant.

**Figure 4:**
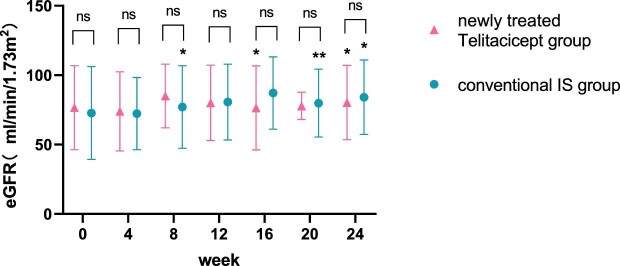
Comparison of eGFR level between the newly treated telitacicept subgroup and the conventional IS group. The dots represent the mean value of eGFR and the bars represent the SD. The level of eGFR was compared with the baseline level every 4 weeks. *P < .05, **P < .01; ns: not significant.

The level of eGFR at 24 weeks increased from 70.10 ± 32.88 ml/min/1.73 m^2^ to 71.21 ± 31.49 ml/min/1.73 m^2^ (*P* = .065) in the whole telitacicept group, from 76.58 ± 30.26 ml/min/1.73 m^2^ to 80.30 ± 26.76 ml/min/1.73 m^2^ (*P* = .016) in the newly treated telitacicept subgroup and from 72.73 ± 33.41 ml/min/1.73 m^2^ to 84.08 ± 26.81 ml/min/1.73 m^2^ (*P* = .011) in the conventional IS group. There were no statistically significant differences between the newly treated telitacicept subgroup and the conventional IS group in terms of eGFR at baseline (*P* = .684), 4 weeks (*P* = .844), 8 weeks (*P* = .419), 12 weeks (*P* = .945), 16 weeks (*P* = .308), 20 weeks (*P* = .792) and 24 weeks (*P* = .783) (Figs. 3 and 4).

### Secondary outcomes

**Figure 5:**
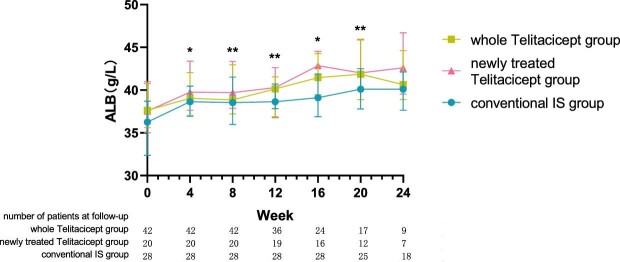
The changes in ALB level in the whole telitacicept group, the newly treated telitacicept subgroup and the conventional IS group during each follow-up period. The dots represent the median proteinuria values and the bars represent the 25th and 75th percentiles. In the whole telitacicept group, the level of ALB was compared with the baseline level every 4 weeks. *P < .05, **P < .01.

**Figure 6:**
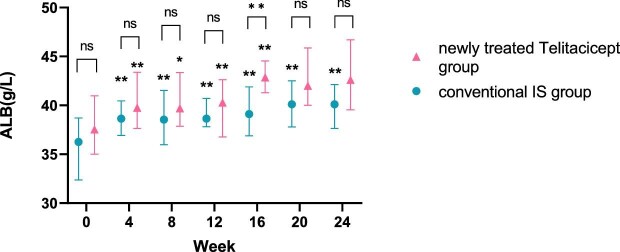
Comparison of ALB level between the newly treated telitacicept subgroup and the conventional IS group. The dots represent the median ALB values and the bars represent the 25th and 75th percentiles. The level of ALB was compared with the baseline level every 4 weeks. *P < .05, **P < .01; ns: not significant.

The level of ALB at 24 weeks increased from 37.65 g/l (IQR 35.60–40.75) to 40.65 g/l (IQR 38.90–44.60) (*P* = .161) in the whole telitacicept group, from 37.55 g/l (IQR 35.00–40.98) to 42.60 g/l (IQR 39.55–46.70) (*P* = .066) in the newly treated telitacicept subgroup and from 36.25 g/l (IQR 32.38–38.70) to 40.10 g/l (IQR 37.65–42.13) (*P* = .001) in the conventional IS group. There were no statistically significant differences between the newly treated telitacicept subgroup and the conventional IS group in terms of ALB level at baseline (*P* = .225), 4 weeks (*P* = .123), 8 weeks (*P* = .185), 12 weeks (*P* = .354), 20 weeks (*P* = .156) and 24 weeks (*P* = .125). The ALB level in the newly treated telitacicept subgroup was significantly higher than in the conventional IS group at 16 weeks (*P* = .002) (Figs. 5 and 6).

**Figure 7:**
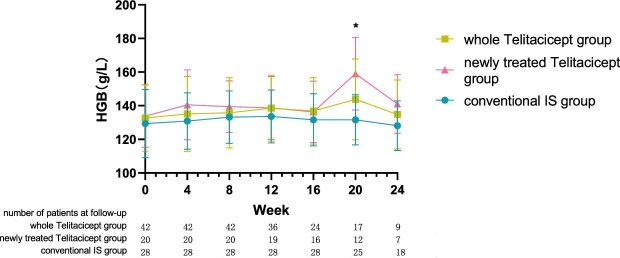
The changes in Hgb level in the whole telitacicept group, the newly treated telitacicept subgroup and the conventional IS group during each follow-up period. The dots represent the mean value of Hgb and the bars represent the SD. In the whole telitacicept group, the level of Hgb was compared with the baseline level every 4 weeks. *P < .05.

**Figure 8:**
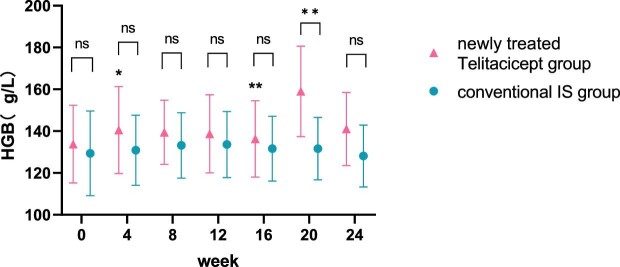
Comparison of Hgb level between the newly treated telitacicept subgroup and the conventional IS group. The dots represent the mean value of Hgb and the bars represent the SD. The level of Hgb was compared with the baseline level every 4 weeks. *P < .05, **P < .01; ns: not significant.

The level of Hgb at 24 weeks increased from 132.67 ± 19.79 g/l to 134.78 ± 20.48 g/l (*P* = .111) in the whole telitacicept group and from 133.80 ± 18.61 g/l to 141.00 ± 17.42 g/l (*P* = .111) in the newly treated telitacicept subgroup, while it decreased from 129.36 ± 20.27 g/l to 128.12 ± 14.79 g/l (*P* = .601) in the conventional IS group. There were no statistically significant differences between the newly treated telitacicept subgroup and the conventional IS group in terms of Hgb level at baseline (*P* = .443), 4 weeks (*P* = .112), 8 weeks (*P* = .242), 12 weeks (*P* = .383), 16 weeks (*P* = .455) and 24 weeks (*P* = .115). The Hgb level in the newly treated telitacicept subgroup was significantly higher than in the conventional IS group at 20 weeks (*P* = .006) (Figs. 7 and 8).

**Figure 9:**
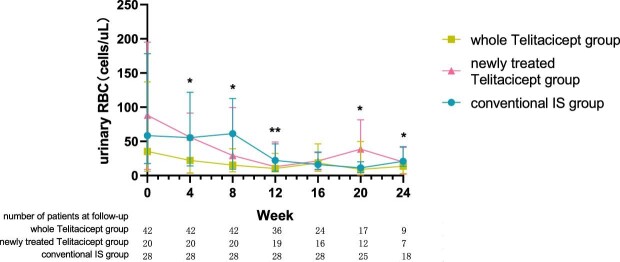
The changes in urinary RBC count in the whole telitacicept group, the newly treated telitacicept subgroup and the conventional IS group during each follow-up period. The dots represent the median urinary RBC counts and the bars represent the 25th and 75th percentiles. In the whole telitacicept group, the urinary RBC count was compared with the baseline level every 4 weeks. *P < .05, **P < .01.

**Figure 10:**
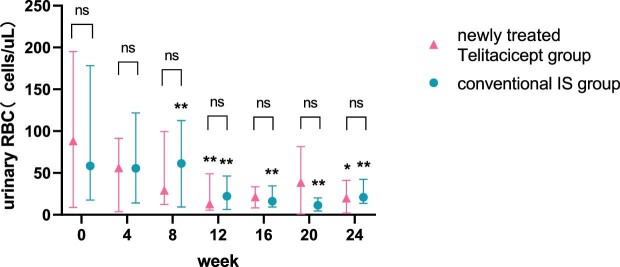
Comparison of urinary RBC count between the newly treated telitacicept subgroup and the conventional IS group. The dots represent the median urinary RBC counts and the bars represent the 25th and 75th percentiles. The urinary RBC count was compared with the baseline level every 4 weeks. *P < .05, **P < .01; ns: not significant.

The urinary RBC count at 24 weeks decreased from 35.40 cells/μl (IQR 6.23–137.08) to 13.60 cells/μl (IQR 3.00–22.10) (*P* = .015) in the whole telitacicept group, from 88.15 cells/μl (IQR 8.85–195.03) to 19.80 cells/μl (IQR 1.95–41.05) (*P* = .043) in the newly treated telitacicept subgroup and from 58.45 cells/μl (IQR 17.60–178.28) to 21.00 cells/μl (IQR 13.60–42.30) (*P* = .002) in the conventional IS group. There were no statistically significant differences between the newly treated telitacicept subgroup and the conventional IS group in terms of urinary RBC count at baseline (*P* = .818), 4 weeks (*P* = .248), 8 weeks (*P* = .456), 12 weeks (*P* = .785), 16 weeks (*P* = .714), 20 weeks (*P* = .968) and 24 weeks (*P* = .456) (Figs. 9 and 10).

### Treatment efficacy

There were no statistically significant differences in efficacy rates among the three groups at 4 weeks (*P* = .685), 8 weeks (*P* = .765), 12 weeks (*P* = .382), 16 weeks (*P* = .325), 20 weeks (*P* = .303) and 24 weeks (*P* = .503) (Table 2). At the last follow-up of each patient, the whole telitacicept group had 19 patients who had achieved PR, 9 patients who had achieved CR and an overall efficacy rate of 66.7%. In the newly treated telitacicept subgroup there were 12 patients who had achieved PR, 5 patients who had achieved CR and an overall efficacy rate of 85.0%. Similarly, in the conventional IS group, there were 10 patients who had achieved PR, 11 patients who had achieved CR and an overall efficacy rate of 75.0%. There were no statistically significant differences in efficacy rates among the three groups (*P* = .337) (Table 3).

**Table 2:** Efficacy rates of the three groups during each follow-up period.

Group	Efficacy	4 weeks	8 weeks	12 weeks	16 weeks	20 weeks	24 weeks
Whole telitacicept group (*n* = 42)	Effective, *n* (%)	13 (30.95)	19 (45.24)	24 (66.67)	16 (66.67)	13 (76.47)	7 (77.78)
	Ineffective, *n* (%)	29 (69.05)	23 (54.76)	12 (33.33)	8 (33.33)	4 (23.53)	2 (22.22)
	Total, *n*	42	42	36	24	17	9
Newly treated telitacicept subgroup (*n* = 20)	Effective, *n* (%)	8 (40)	11 (55.00)	16 (84.21)	14 (87.50)	11 (91.67)	7 (100.00)
	Ineffective, *n* (%)	12 (60)	9 (45.00)	3 (15.79)	2 (12.50)	1 (8.33)	0 (0.00)
	Total, *n*	20	20	19	16	12	7
Conventional IS group (*n* = 28)	Effective, *n* (%)	8 (28.57)	14 (50.00)	20 (71.43)	20 (71.43)	17 (68.00)	14 (77.78)
	Ineffective, *n* (%)	20 (71.43)	14 (50.00)	8 (28.57)	8 (28.57)	8 (32.00)	4 (22.22)
	Total, *n*	28	28	28	28	25	18
*P*-value		.685	.765	.382	.325	.303	.503

**Table 3:** Efficacy rates of the three groups at the last follow-up of each patient.

Efficacy, *n* (%)	Whole telitacicept group (*n* = 42)	Newly treated telitacicept subgroup (*n* = 20)	Conventional IS group (*n* = 28)	*P*-value
No remission	14 (33.3)	3 (15.0)	7 (25.0)	
PR	19 (45.2)	12 (60.0)	10 (35.7)	
CR	9 (21.4)	5 (25.0)	11 (39.3)	
Efficacy rate	28 (66.7)	17 (85.0)	21 (75.0)	.337

**Table 4:** Incidence of adverse reactions in the three groups.

Adverse reactions, *n* (%)	Whole telitacicept group (*n* = 42)	Newly treated telitacicept subgroup (*n* = 20)	Conventional IS group (*n* = 28)	*P*-value
Local skin reactions	14 (33.3)	7 (35.0)		
Infection	2 (4.8)	1 (5.0)	2 (7.1)	
Hair loss			7 (25.0)	
Skin acne			4 (14.3)	
Obesity			4 (14.3)	
Hand tremors			2 (7.1)	
Osteoporosis			2 (7.1)	
Fatigue	1 (2.4)	1 (5.0)		
Incidence of AEs	17 (40.5)	9 (45.0)	21 (75.0)	.035

*P*-value was the statistical difference between the newly treated telitacicept subgroup and the conventional IS group.

**Figure 11:**
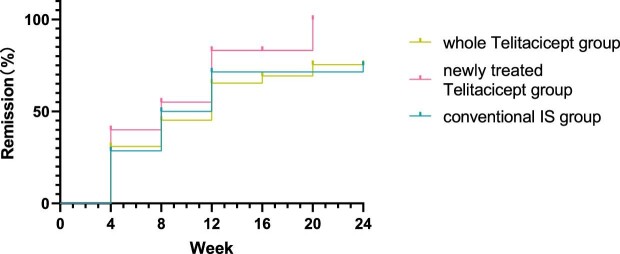
Comparison of the cumulative efficacy rates among the three groups using Kaplan–Meier survival analysis (logrank test, χ^2^ = 2.085, *P* = .35).

Further analysis was conducted using Kaplan–Meier survival analysis to compare the cumulative efficacy rates among the three groups, which showed no statistically significant differences (logrank test, χ^2^ = 2.085, *P* = .35) (Fig. 11).

### Safety and AEs

During the follow-up periods, no SAEs were observed. In the whole telitacicept group, 14 patients experienced local skin reactions, 2 patients had infections and 1 patient experienced fatigue (incidence of AEs was 40.5%). In the newly treated telitacicept subgroup, seven patients experienced local skin reactions, one patient had infection and one patient experienced fatigue (incidence of AEs was 45.0%). In the conventional IS group, seven patients experienced hair loss, four patients had skin acne, four patients developed obesity, two patients had hand tremors, two patients had infections and two patients had osteoporosis (incidence of AEs was 75%). The incidence of AEs in the newly treated telitacicept subgroup is significantly lower than that in the conventional IS group (*P* = .035).

## DISCUSSION

IgAN can manifest in different degrees of symptoms such as haematuria, proteinuria, hypertension and renal failure. If not properly controlled, it can gradually progress to end-stage renal disease. The fundamental cause of IgAN has been extensively studied and is now understood at a submolecular level. At the core of the disease is the presence of immune complexes containing specific O-glycoforms of IgA1 [13, 14]. These complexes play a crucial role in the pathogenesis of IgAN.

Selective inhibitors of B cell activating factor (BAFF) and APRIL have shown promise in treating IgAN. The interim results from the phase 2/3 study BRIGHT-SC (NCT02062684) showed that the BAFF-selective inhibitor blisibimod significantly reduced B cell subsets and Ig levels [15]. Anti-APRIL antibodies such as sibeprenlimab (VIS649) showed reductions in Gd-IgA1, IgA and proteinuria in a phase 2 trial [16]. Another anti-APRIL antibody, Zigakibart (BION-1301), is undergoing a phase 3 trial (NCT05852938). Dual BAFF/APRIL inhibitors such as povetacicept and atacicept are under investigation, with atacicept showing significant reductions in proteinuria and stabilization of eGFR in phase 2b trials [17]. Additionally, Nefecon, a novel gut-targeted oral budesonide formulation, has garnered significant attention. It has shown proven effects not only in reducing proteinuria and stabilizing GFR, but also in lowering Gd-IgA1 levels in treating IgAN [18, 19].

Telitacicept works by binding to BLyS and APRIL, blocking their interaction with B cell receptors and thereby inhibiting the activation and proliferation of B cells [4]. This can reduce inflammation and damage in the process of autoimmune reactions, improving symptoms of related autoimmune diseases [20]. In addition to its regulatory effects on B cells, telitacicept can also influence certain types of T cells and macrophages, which play important roles in immune responses. By modulating the function of these immune cells, telitacicept can achieve overall immune regulation [21, 22]. The therapeutic efficacy of telitacicept in the treatment of SLE [7, 23, 24] has been proven. The use of telitacicept in treating IgAN has shown promising results [11, 25, 26], but its effects may vary across different ethnic groups and need further investigation. Case reports by Zhang *et al.* [27] have shown successful treatment of refractory membranous nephropathy with telitacicept, achieving CR in patients treated with telitacicept alone. Research by Ding *et al.* [28] has indicated that telitacicept following plasma exchange has the potential to be a safe treatment for patients with recurrent neuromyelitis optica spectrum disorders.

This study analysed the efficacy and safety of telitacicept therapy for IgAN and compared the treatment efficacy between telitacicept and conventional IS therapy. The results of our study showed that telitacicept achieved similar clinical efficacy in the treatment of IgAN, significantly reducing proteinuria and increasing eGFR. Patients treated with the addition of telitacicept experienced a rapid response in the reduction of proteinuria. The CS exposure was less in the newly treated telitacicept subgroup, yet it still led to a non-inferior efficacy rate, with initial doses being lower and more rapidly tapered. Furthermore, the incidence of AEs was lower for telitacicept compared with conventional IS therapy. Telitacicept can produce rapid treatment efficacy, with a significant reduction in 24-hour proteinuria and a significant increase in ALB levels after 4 weeks of treatment. The degree of reduction in 24-hour proteinuria in the newly treated telitacicept subgroup during the first 12 weeks of treatment seemed more pronounced compared with the conventional IS group, suggesting a faster onset of effect for telitacicept. However, there were no statistically significant differences between the two groups at each follow-up period, which may be attributed to the large data variability and insufficient sample size. Patients in the whole telitacicept group showed stable renal function and patients in the newly treated telitacicept subgroup showed a significant increase in eGFR. At 24 weeks, the newly treated telitacicept subgroup showed a more pronounced decrease in 24-hour proteinuria compared with the conventional IS group. Hgb levels showed an increasing trend in the whole telitacicept group and the newly treated telitacicept subgroup, but a decreasing trend was observed in the conventional IS group. These trends in changes require further long-term follow-up observations. The efficacy rates of the three groups during follow-up periods showed no statistically significant differences, indicating that telitacicept therapy is not inferior to conventional IS therapy in treating IgAN.

In 42 cases of telitacicept application, the majority of AEs were local skin reactions at the injection site, with some patients experiencing mild itching and swelling. These reactions typically resolved on their own within 2–3 days after medication and mostly occurred within 4–8 weeks after the initial dose. Beyond 8 weeks, with increased medication doses, the severity of local skin reactions gradually decreased or even ceased to occur. Two patients experienced infections, including one case of herpes zoster and one case of pneumonia. After treatment, these patients recovered well. One patient experienced fatigue, but this symptom significantly improved after reducing the dosage from 240 mg to 160 mg once a week in SC injections. Overall, no SAEs were observed in patients treated with telitacicept. Compared with conventional IS therapy, telitacicept showed better safety.

The above results demonstrate that the efficacy of telitacicept is comparable to that of patients treated with conventional IS therapy, indicating that telitacicept alone or in combination with CS therapy or IS agents may effectively delay the progression of IgAN. These findings suggest that telitacicept may be a supplement or alternative treatment to CS, thereby reducing the need for CS and mitigating associated side effects.

This study has some limitations. It is a single-centre, retrospective study, inevitably subject to selection bias. Some patients treated with telitacicept were also co-treated with CS or IS agents, which decreased the difference between the newly treated telitacicept subgroup and the conventional IS group. The levels of Gd-IgA1 were not assessed before and during treatment. Due to the impact of the COVID-19 pandemic, some patients were unable to return to the hospital on time for follow-up visits, resulting in missing values. In addition, the sample size of this study is small and the results need to be further verified by larger, multicentre studies.

## CONCLUSIONS

Compared with conventional IS therapy, telitacicept achieved similar clinical efficacy in the treatment of IgAN, significantly reducing proteinuria and increasing eGFR while exhibiting good safety. Telitacicept may be a supplement or alternative treatment for IgAN, helping to avoid or minimize the side effects associated with CS and providing a novel therapeutic option for IgAN treatment.

## Supplementary Material

sfae285_Supplemental_File

## Data Availability

The data underlying this article will be shared upon reasonable request to the corresponding author.

## References

[1] Magistroni R, D'Agati VD, Appel GB et al. New developments in the genetics, pathogenesis, and therapy of IgA nephropathy. Kidney Int 2015;88:974–89. 10.1038/ki.2015.252 26376134 PMC4653078

[2] Zhao N, Hou P, Lv J et al. The level of galactose-deficient IgA1 in the sera of patients with IgA nephropathy is associated with disease progression. Kidney Int 2012;82:790–6. 10.1038/ki.2012.197 22673888 PMC3443545

[3] Cheung CK, Barratt J, Liew A et al. The role of BAFF and APRIL in IgA nephropathy: pathogenic mechanisms and targeted therapies. Front Nephrol 2023;3:1346769. 10.3389/fneph.2023.1346769 38362118 PMC10867227

[4] Sallustio F, Curci C, Chaoul N et al. High levels of gut-homing immunoglobulin A^+^ B lymphocytes support the pathogenic role of intestinal mucosal hyperresponsiveness in immunoglobulin A nephropathy patients. Nephrol Dial Transplant 2021;36:452–64. 10.1093/ndt/gfaa264 33200215 PMC7898021

[5] Dhillon S . Telitacicept: first approval. Drugs 2021;81:1671–5. 10.1007/s40265-021-01591-1 34463932

[6] Fan Y, Gao D, Zhang Z. Telitacicept, a novel humanized, recombinant TACI-Fc fusion protein, for the treatment of systemic lupus erythematosus. Drugs Today (Barc) 2022;58:23–32. 10.1358/dot.2022.58.1.3352743 35107091

[7] Chen Y, Shi N, Lei X et al. The efficacy of rituximab plus belimumab or telitacicept in refractory lupus nephritis. Rheumatology (Oxford) 2023;kead674. 10.1093/rheumatology/kead674 38145455

[8] Li S, Deng S, Wen S et al. Telitacicept treatment refractory lupus nephritis: a case report. Case Rep Nephrol Dial 2024;14:42–7. 10.1159/000538033 38524729 PMC10959545

[9] Jin HZ, Li YJ, Wang X et al. Efficacy and safety of telitacicept in patients with systemic lupus erythematosus: a multicentre, retrospective, real-world study. Lupus Sci Med 2023;10:e001074. 10.1136/lupus-2023-001074 38007228 PMC10679987

[10] Xu D, Fang J, Zhang S et al. Efficacy and safety of telitacicept in primary Sjogren's syndrome: a randomized, double-blind, placebo-controlled, phase 2 trial. Rheumatology (Oxford) 2024;63:698–705. 10.1093/rheumatology/kead265 37399108

[11] Lv J, Liu L, Hao C et al. Randomized phase 2 trial of telitacicept in patients with IgA nephropathy with persistent proteinuria. Kidney Int Rep 2023;8:499–506. 10.1016/j.ekir.2022.12.014 36938094 PMC10014376

[12] Levey AS, Stevens LA, Schmid CH et al. A new equation to estimate glomerular filtration rate. Ann Intern Med 2009;150:604–12. 10.7326/0003-4819-150-9-200905050-00006 19414839 PMC2763564

[13] Pattrapornpisut P, Avila-Casado C, Reich HN. IgA nephropathy: Core Curriculum 2021. Am J Kidney Dis 2021;78:429–41. 10.1053/j.ajkd.2021.01.024 34247883

[14] Chang S, Li XK. The role of immune modulation in pathogenesis of IgA nephropathy. Front Med 2020;7:92. 10.3389/fmed.2020.00092 PMC7105732 32266276

[15] Xin H, Gaosi X. An update on targeted treatment of IgA nephropathy: an autoimmune perspective. J Front Pharmacol 2021;12:715253. 10.3389/fphar.2021.715253 PMC8419281 34497518

[16] Mathur M, Barratt J, Chacko B et al. A phase 2 trial of sibeprenlimab in patients with IgA nephropathy. N Engl J Med 2024;390:20–31. 10.1056/NEJMoa2305635 37916620 PMC7615905

[17] Lafayette R, Barbour S, Israni R et al. A phase 2b, randomized, double-blind, placebo-controlled, clinical trial of atacicept for treatment of IgA nephropathy. Kidney Int 2024;105:1306–15. 10.1016/j.kint.2024.03.012 38552841

[18] Lafayette R, Kristensen J, Stone A et al. Efficacy and safety of a targeted-release formulation of budesonide in patients with primary IgA nephropathy (NefIgArd): 2-year results from a randomised phase 3 trial. Lancet 2023;402:859–70. 10.1016/S0140-6736(23)01554-4 37591292

[19] Barratt J, Lafayette R, Kristensen J et al. Results from part A of the multi-center, double-blind, randomized, placebo-controlled NefIgArd trial, which evaluated targeted-release formulation of budesonide for the treatment of primary immunoglobulin A nephropathy. Kidney Int 2023;103:391–402. 10.1016/j.kint.2022.09.017 36270561

[20] Shi F, Xue R, Zhou X et al. Telitacicept as a BLyS/APRIL dual inhibitor for autoimmune disease. Immunopharmacol Immunotoxicol 2021;43:666–73. 10.1080/08923973.2021.1973493 34519594

[21] Cai J, Gao D, Liu D et al. Telitacicept for autoimmune nephropathy. Front Immunol 2023;14:1169084. 10.3389/fimmu.2023.1169084 37342346 PMC10277628

[22] Cai S, Hu Z, Chen Y et al. BLyS/APRIL dual inhibition for IgG4-RD: a prospective single-arm clinical trial of telitacicept. Ann Rheum Dis 2023;82:881–3. 10.1136/ard-2022-223529 36657954 PMC10313947

[23] Wu D, Li J, Xu D et al. Telitacicept in patients with active systemic lupus erythematosus: results of a phase 2b, randomised, double-blind, placebo-controlled trial. Ann Rheum Dis 2024;83:475–87. 38129117 10.1136/ard-2023-224854 PMC10958275

[24] Sun L, Shen Q, Gong Y et al. Safety and efficacy of telitacicept in refractory childhood-onset systemic lupus erythematosus: a self-controlled before-after trial. Lupus 2022;31:998–1006. 10.1177/09612033221097812 35499216

[25] Wu L, Du X, Lu X. Role of telitacicept in the treatment of IgA nephropathy. Eur J Med Res 2023;28:369. 10.1186/s40001-023-01320-2 37737205 PMC10515419

[26] Liang D, Li X. Efficacy and safety of telitacicept in patients with IgA nephropathy. Pak J Med Sci 2023;39:1897. 10.12669/pjms.39.6.8393 37936774 PMC10626081

[27] Zhang L, Jin H, Wang D et al. Case report: successful treatment of refractory membranous nephropathy with telitacicept. Front Immunol 2023;14:1268929. 10.3389/fimmu.2023.1268929 37915584 PMC10616774

[28] Ding J, Cai Y, Deng Y et al. Telitacicept following plasma exchange in the treatment of subjects with recurrent NMOSD: study protocol for a single-center, single-arm, open-label study. Front Neurol 2021;12:596791. 10.3389/fneur.2021.596791 33868140 PMC8044936

