# Supplementary material for: Efficacy and safety of telitacicept, a BLyS/APRIL dual inhibitor, in the treatment of IgA nephropathy: a retrospective case–control study
Source: Clin Kidney J. 2024 Sep 13;17(10):sfae285. doi: 10.1093/ckj/sfae285 (PMC11464987; doi:10.1093/ckj/sfae285)
Supplement: sfae285_Supplemental_File [file sfae285_supplemental_file.pdf]

Supplementary data

	whole Telitacicept group（n=42）	newly treated Telitacicept subgroup（n=20）	conventional IS group（n=28）	*P*-value
previous CS, n（%）	16 (38.1%)	0 (0%)	0 (0%)	
previous CS treatment regimen, n（%）	5 (11.9%): MP 120-250 mg/d × 3d, followed by Pred 40-45 mg/d; 11 (26.2%): Pred 40 (30-40) mg/d			
previous IS agents, n（%）	15 (35.7%)	0 (0%)	0 (0%)	
previous IS agents treatment regimen, n（%）	8 (19.0%): TPE; 2 (4.8%): CTX; 2 (4.8%): MMF; 2 (4.8%): LEF; 1 (2.4%): CsA			
current CS or IS agents, n（%）	22 (52.4%)	10 (50%)	28 (100.0%)	<0.01
current CS, n（%）	16 (38.1%)	8 (40.0%)	28 (100%)	<0.01
current CS	3 (7.1%): MP	2 (10.0%): MP 80	6 (21.4%):MP	

treatment regimen, n（%）	80-120 mg/d × 3d, followed by Pred 30-40 mg/d; 13 (31.0%): Pred 30 (20-40) mg/d	mg/d × 3d, followed by Pred 30 mg/d; 6 (30.0%): Pred 30 mg/d	120-240 mg/d × 3d, followed by Pred 40-50 mg/d; 22 (78.6%): Pred 40 (30-50) mg/d	
current IS agents，n（%）	16 (38.1%)	7 (35%)	13 (46.4%)	0.311
current IS agents treatment regimen, n（%）	11 (26.2%): TPE; 3 (7.1%): LEF; 2 (4.8%): MMF	3 (15.0%): TPE; 3 (15.0%): LEF; 1 (5.0%): MMF	11 (39.3%): CTX (CTX accumulated to 5 [4.8-6] g); 1 (3.6%): LEF; 1 (3.6%): MMF;	

Table S1. The specific usage of CS and IS agents in the three groups. *P*-value was the statistical difference between the newly treated Telitacicept subgroup and the conventional IS group; MP, Methylprednisolone; Pred, Prednisone; TPE, Tripterygium preparations; CTX, Cyclophosphamide; MMF, Mycophenolate Mofetil; LEF, Leflunomide; CsA, Cyclosporine

In the whole Telitacicept group, 16 (38.1%) patients were treated with CS before starting Telitacicept therapy. Five patients received Methylprednisolone at 120-250 mg daily for three consecutive days, followed by Prednisone at 40-45 mg daily, while 11 patients were treated with Prednisone at 40 (30-40) mg daily. CS treatment lasted for 10 (6-12) months and was discontinued 8 (3-20) months before starting Telitacicept therapy. Additionally, 15 (35.7%) patients were treated

with IS agents before starting Telitacicept therapy, including Leflunomide in two patients, Mycophenolate Mofetil in two patients, Cyclophosphamide in two patients, Tripterygium preparations in eight patients, and Cyclosporine in one patient. IS agents were used for 8 (3-12) months and discontinued 10 (3-27) months before starting Telitacicept therapy. Upon entering the study, 22 (52.4%) patients received CS or IS agents. Of these, 16 (38.1%) patients were treated with CS. Three patients received Methylprednisolone at 80-120 mg daily for three consecutive days, followed by Prednisone at 30-40 mg daily, and 13 patients were treated with Prednisone at 30 (20-40) mg daily. Furthermore, 16 (38.1%) patients were treated with IS agents when entering the study, including Leflunomide in three patients, Mycophenolate Mofetil in two patients, and Tripterygium preparations in 11 patients.

In the newly treated Telitacicept subgroup, 10 (50%) patients started treatment with CS or IS agents alongside Telitacicept upon entering the study. Of these, 8 (40.0%) patients were treated with CS. Two patients received Methylprednisolone at 80 mg daily for three consecutive days, followed by Prednisone at 30 mg daily, and six patients were treated with Prednisone at 30 mg daily. Prednisone tapering started after 4-6 weeks, reducing the dose by 5mg every 2-4 weeks. Additionally, 7 (35%) patients were treated with IS agents when entering the study, including Leflunomide in three patients, Mycophenolate Mofetil in one patient, and Tripterygium preparations in three patients.

In the conventional IS group, 28 (100.0%) patients were included in the study from the start of IS treatment. Six patients received Methylprednisolone at 120-240 mg daily for three consecutive days, followed by Prednisone at 40-50 mg daily, and 22 patients were treated with Prednisone at 40 (30-50) mg daily. 13 (46.4%) patients were treated with IS agents when entering

the study, including Leflunomide in one patient, Mycophenolate Mofetil in one patient, and Cyclophosphamide in 11 patients (Cyclophosphamide accumulated to 5 [4.8-6] g).

Among patients treated with Telitacicept, the treatment course of some patients is worth paying attention to. A 40-year-old male with proteinuria and elevated serum creatinine (Scr) for seven years, had previously been diagnosed with IgAN Lee III-IV (M1E0S1T1-C0) and obesity-related glomerulopathy. Due to obesity, the patient had refused CS therapy but was given Telitacicept, at a dose of 160 mg/week SC injections and ARB, along with active weight loss management. CR was achieved after 4 weeks of treatment (proteinuria value at the start of Telitacicept treatment was 1.059g/d). A 32-year-old female with proteinuria for two years, previously diagnosed with IgAN Lee III-IV (M1E0S1T0-C1), refused IS agents and ARB treatment due to pregnancy plans and was given Telitacicept at a dose of 160 mg/week SC injections in combination with CS therapy. PR was achieved after 12 weeks, and CR after 24 weeks (proteinuria value at the start of Telitacicept treatment was 1.057g/d). A 29-year-old male with proteinuria for three years, elevated Scr levels for 2.5 years, and a 15-year history of psoriasis, previously diagnosed with IgAN Lee V (M0E0S1T2-C0), had previously received CS and cyclophosphamide pulse therapy without CR. Later, with Telitacicept alone at a dose of 160 mg/week SC injections, CR was achieved after 4 weeks of treatment (proteinuria value at the start of Telitacicept treatment was 0.531g/d). The psoriasis symptoms also improved significantly during the treatment, though the mechanism behind this improvement requires further exploration. A 24-year-old male with IgAN Lee II-III (M0E1S1T0-C1) diagnosed three years ago, had to stop all CS therapy and IS agents due to abnormal liver function. He then continued with only Telitacicept at a dose of 160 mg/week SC injections and achieved PR after 8 weeks of treatment

(proteinuria value at the start of Telitacicept treatment was 1.921g/d). A 20-year-old male with IgAN Lee V (M1E1S1T1-C1) was initially treated with CS, MMF, hydroxychloroquine, and ARB, but CR was not achieved. Later, Telitacicept at a dose of 160 mg/week SC injections in combination with ARB was given, and CR was achieved after 4 weeks (proteinuria value at the start of Telitacicept treatment was 0.734g/d). A 22-year-old female with IgAN Lee III (M1E1S1T0-C0) diagnosed one year ago, was given Telitacicept at a dose of 160 mg/week SC injections in combination with hydroxychloroquine and ARB. PR was achieved after 4 weeks, and CR was achieved after 16 weeks (proteinuria value at the start of Telitacicept treatment was 1.946g/d). These successful cases of treatment have given us full confidence in using Telitacicept to treat IgAN.
